# Use of Teleconsultations in a Regional Stereotactic Radiosurgery Service: Pilot Study

**DOI:** 10.2196/15598

**Published:** 2021-02-05

**Authors:** Micheal O'Cathail, Luis Aznar-Garcia, Ananth Sivanandan, Claire Diver, Poulam Patel, Pui-Shan Tang, Judith Christian

**Affiliations:** 1 Department of Oncology & Radiotherapy Nottingham University Hospital NHS Trust Nottingham United Kingdom; 2 School of Medicine University of Nottingham Nottingham United Kingdom; 3 School of Medicine & Health Sciences University of Nottingham Nottingham United Kingdom; 4 East Midlands Academic Health Science Network Nottingham United Kingdom

**Keywords:** telemedicine, teleconsultations, brain metastases, stereotactic radiosurgery, mobile phone

## Abstract

**Background:**

The National Health Service Long Term Plan details plans to make digital interactions available to all patients in 5 years. Teleconsultations can improve access to specialist services; however, there is a lack of evidence for the use of teleconsultations in an oncology setting in the United Kingdom.

**Objective:**

We aim to describe a service evaluation of teleconsultations for patients attending a regional brain metastases clinic. These patients have unique travel restrictions that prevent them from driving.

**Methods:**

From April to October 2018, all patients attending the brain metastases clinic were offered the choice of teleconsultation in place of a face-to-face appointment. Feedback was assessed using a satisfaction questionnaire, and data of all clinic attendances were collected.

**Results:**

A total of 69 individual patients had 119 appointments over the duration of the pilot, of which 36 (30.2%) were new patient appointments and 73 (61.3%) were follow-ups. Of the 69 patients, 24 (35%) took part in teleconsultations (41/119, 34.5%). User satisfaction was high, and no patients who took part in a teleconsultation reverted to face-to-face appointments. These patients avoided 2521 miles (61.6 miles per appointment) of hospital-associated travel and travel costs of £441.48 (US $599.83) to £10.78 (US $14.65) per appointment.

**Conclusions:**

Teleconsultations appear to be acceptable in this cohort of patients with brain metastases attending a regional stereotactic radiosurgery service with the potential for significant savings in travel and expenses.

## Introduction

### Overview

As part of its “Five Year Forward View” in 2014 [1], National Health Service (NHS) England recognized the changing needs of patients and the need to capitalize on the opportunities that new technologies present. This view was reinforced in the recently announced “Long Term Plan” that aims to give all patients the choice of technology-enabled consultations, including the use of video consultations within the next 5 years [2].

The UK’s National Information Board and the Royal College of Physicians suggest that traditional models of outpatient care are outdated in the 21st century. Therefore, the NHS needs to embrace technology and offer patients alternate ways of interacting with the NHS [3,4].

Health care is becoming increasingly specialized, with small hospitals now unable to provide a full range of health care service. Specialized services are therefore provided by larger institutions thus creating a *hub and spoke* model of health care [5]. In some services, such as specialized surgery and cancer care, this has been shown to improve the quality of care for patients [1]. However, this model may be quite burdensome on patients as it requires travel [6]. Telemedicine can reduce travel and improve access to such specialist services by bringing care to patients [7].

Telemedicine, as defined by Sood et al [8], is “The use of communications networks for delivery of health care services and medical education from one geographical location to another, primarily to address challenges like uneven distribution and shortage of infrastructural and human resources.” The NHS uses *technology-enabled care* to describe digital patient interactions and *teleconsultations* for video consultations [9].

### What Are Brain Metastases?

Brain metastases are secondary brain tumors that spread to the brain from other parts of the body. These patients may benefit from treatment with stereotactic radiosurgery (SRS), which is a specialized form of radiotherapy that can improve survival when compared with whole brain radiotherapy [10]. At present, this is only offered in a few regional centers in the United Kingdom [11]. The East Midlands brain metastases service was established in 2018 to improve patient access to this treatment in the region. Before its establishment, it was demonstrated that referrals for SRS were inconsistent, which meant that there was inequitable access to specialist care [12]. Patients with brain metastases face unique difficulties in attending hospital appointments owing to the fact that their diagnosis precludes them from driving [13]. They rely on family, public transport, or hospital transport to reach the hospital. The regional nature of our service makes this even more difficult. Therefore, a teleconsultation service was proposed with the aim of improving access to specialist care and reducing patient travel burden.

### Current Evidence and International Experience

There is sparse evidence in the United Kingdom for the use of teleconsultations in a UK cancer care setting [14] and none specifically in radiotherapy care. A scoping review of the current evidence in the United Kingdom concluded that teleconsultations are safe and generally well received by patients across a broad variety of clinical settings but that they should be offered as a choice rather than a replacement of face-to-face appointments [15]. They and other authors concluded that the introduction of teleconsultation should be reviewed after a set period with service evaluations and feedback from stakeholders [14,15].

Teleconsultations in oncology have long been used in health services in Australia and Canada where burdensome traveling for patients and physicians and accessibility are issues [16,17]. In Australia, the distances that patients and staff have to travel to have a face-to-face appointment were so large that they would have to fly to make it practical. By implementing a teleconsultation service, they have demonstrated that safe oncological care can be provided while delivering a high-quality patient experience, with high rates of satisfaction. In addition, a teleconsultation service can realize significant cost savings for health care providers and patients alike [17-19]. Although this is not a likely scenario in the United Kingdom, some patients have to travel up to 5 hours to see the specialist SRS team.

### Study Aim

The aim of this pilot study is to evaluate patient acceptability and satisfaction with the use of teleconsultations in the assessment and management of patients with brain metastases undergoing SRS and to describe the proportion of clinical activity. We aim to establish whether there are objective demographic differences among those who choose teleconsultations.

## Methods

### Study Participants

From April to October 2018, patients attending the regional brain metastases service in our center were offered the choice of having a face-to-face appointment or having a teleconsultation. To be able to take part in a teleconsultation, the patient had to have access to a device capable of supporting video calling (smartphone, tablet, laptop, or desktop computer with webcam) and an email address. A proprietary teleconsultation solution accredited for use in the NHS was used (Medio.link Involve Visual Collaboration Limited).

### Clinical Setting

The East Midlands brain metastases service was established to assess and manage patients undergoing SRS. Cases were discussed at the regional brain metastases multidisciplinary team meeting, the rationale for which has been previously described [12], to determine technical suitability for SRS. Technically suitable patients were reviewed in the brain metastases clinic to ensure clinical suitability for treatment with SRS. All patients were offered the choice of attending either in person or by a teleconsultation link. Irrespective of their choice, they were seen during the same Wednesday afternoon clinic session. A separate clinic code was set up for each modality to record the attendances to each. Participants were free to switch appointment modalities for future appointments. Two physicians were involved in conducting both the teleconsultations and face-to-face clinic assessments, whereas a third was involved in face-to-face clinic assessments only. All 3 physicians covered the same patient group and thus access to the following treatment: patients were reviewed at 1 month and 3 months after SRS and then every 3 months for the first year following treatment.

Following the consultation, patients were asked to fill a feedback questionnaire that was administered through a web-based electronic survey service.

### Feedback Questionnaire

A questionnaire, which was derived from previous studies, was designed. It assessed the use of teleconsultations in an outpatient oncology setting. This included questions on (1) basic demographics (age and gender), (2) distance to the regional center, expected transport mode (if they had attended in person), and appointment type and duration, (3) type of internet connection and device used, (4) patient reported costs of their chosen appointment modality, (5) ranked patient reported benefit of teleconsultations (only if they indicated a preference for these), and (6) 9 statements regarding satisfaction (questions 9-16 and question 18) with different aspects of their consultation were included. Statements were recorded on a 5-point Likert scale with *1* indicating strong disagreement, *3* indicating no opinion, and *5* indicating strong agreement. Question 21 was left as free text to allow patients to suggest future improvements to the service. The satisfaction questions are taken from the previous studies, which has been mentioned earlier, exploring teleconsultations in oncology patients [16,17]. The final question (question 21) asked participants to complete an open-ended feedback question.

The questionnaire was sent to those patients who gave verbal consent at the time of the teleconsultation or confirmation by email afterward and none declined. A reminder was sent (via the e-survey website) a week later if the questionnaire was not completed. Although they may have had more than one appointment during the pilot, participants were asked to complete the questionnaire only once.

A similar feedback questionnaire was administered to patients who attended a face-to-face appointment for comparison. This was given by the clinic support worker when participants arrived for their appointment. Consent was assumed if the questionnaire was returned. A box for survey returns was placed on the reception desk. No reminder was provided to the patients who had face-to-face appointments. The questions are shown in Multimedia Appendix 1.

### Patient and Public Involvement

Before implementing our teleconsultation service, participation in a preclinical pilot was sought from patient volunteers in a local cancer recovery group. In total, 4 participants took part in a test teleconsultation to provide feedback on the video platform’s joining instructions and the quality of the video and audio streams. They were also provided with the proposed questionnaire to the pilot to assess local validity. Minor changes were made to the joining instructions; however, no amendments to the questionnaire were necessary. Following this, we established a teleconsultation service as an option to those attending the brain metastases clinic in April 2018. Here, we describe the results from our 6-month pilot.

### Clinic Attendee Metrics

Anonymized demographic data were collected from all clinic attendances during the pilot duration. This included age, gender, distance, and travel time from the hospital based on the home postcode (fastest route planned by Google Maps), number of attendances, and appointment type (new consultation or follow-up). Appointment costs were estimated based on the distance of the return journey, an average fuel price of £1.28 (US $1.74) per liter (UK average during the pilot) [20] on the assumption that all attendees came by private car with a fuel economy of 51.7 mpg (average new car fuel efficiency in 2017) [21] and car parking costs of £4 (US $5.45) per attendance (based on the cost of trust parking for 1 hour).

### Rationale for Study Design

The NHS, in its *technology-enabled care* guidance, recognizes that randomized controlled trials, though valuable, may prove too costly to be practical for evaluating changes in service provision. Therefore, a more pragmatic method of assessment is a service development review in which the technology can be assessed in practice, supported by patient feedback with the aim of assessing acceptability and satisfaction among users of the service [9]. The Healthcare Quality Improvement Partnership states that a service change is introduced based on evidence that exists in other health and social care settings that have evaluated the service change that these new service developments should be evaluated locally [22]. Moreover, Finch et al [23] found that the evaluation of telemedical services requires more flexible approaches to evidence production than those permitted within the rigid construct of controlled study designs, which may not be reproducible in the real world.

### Data Handling and Analysis

Data were input and stored in an Excel (Microsoft Corporation) file, and descriptive data were generated for each variable. Statistical analyses were performed using GraphPad Prism 7 (GraphPad Software, Inc.). An independent *t* test was used for normally distributed continuous variables. Mann-Whitney *U* test was used for continuous variables that were not normally distributed. Chi-square test was used to determine whether there was an association between categorical variables.

### Ethical Statement

The scope of this service evaluation was approved by and carried out under the scrutiny of the Audit Office of Nottingham University Hospital NHS Trust. According to the Health Research Authority’s decision tool, this project did not require NHS Research Ethics Committee approval.

## Results

### Study Groups

**Figure 1:**
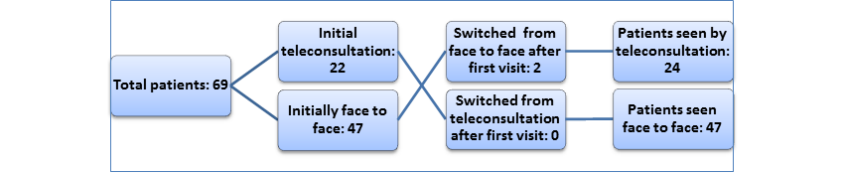
Patient choice of consultation.

In total, 69 individual patients had 119 appointments over the duration of the pilot. Of these, 30.2% (36/119) were new patient appointments and 61.3% (73/119) were follow-ups. In all, 65.5% (78/119) of the appointments were face-to-face and 34.5% (41/119) were teleconsultations. Clinic medium participation is illustrated in Figure 1. Two participants switched after their first face-to-face appointment (both new appointments) to teleconsultations for follow-up.

### Clinic Attendee Metrics

There were several differences between the 2 study groups. Participants in the teleconsultation group was more likely to live further from the hospital and were younger, with a median age of 59 years compared with 65 years in the face-to-face group. The proportion of new consultations was slightly higher in the face-to-face group (26/47, 55%) than in the teleconsultation group (10/22, 45%). There were 78 attendances in the face-to-face group with 40% (19/47) of patients having at least 2 appointments. In the teleconsultation group, there were 41 attendances, with 54% (13/41) of the patients having at least 2 appointments. These are outlined in Table 1.

**Table 1:** Classification of the study participants (N=69) based on their appointment choice and clinic attendance metrics.

Characteristic and Metric					Face to face		Teleconsultation		*P* value
Total patients who chose each consultation type^a^ (N=71), n (%)					47 (66)		24 (34)		N/A^b^
**Sex, n (%)**									
		Male		17 (36)		11 (46)			.43^c^
		Female		30 (64)		13 (54)			.43^c^
	Median age (years; range)				65 (23-84)		59 (32-88)		<.001^d^
**Clinic attendance metrics**									
	**Patient choice for initial consultation (N=69), n (%)**				47 (100)		22 (100)		
		New consultation			26 (55)		10 (45)		.44^c^
		Follow-up consultation			21 (45)		12 (55)		.44^c^
	**Return journey metrics (per appointment)**								
		Median distance, miles (range)			33 (5.2-82)		61.6 (8-130.6)		<.001^e^
		Median estimated travel time^f^, minutes (range)			78 (21-144)		109^g^ (30-198)		<.001^e^
		Median estimated travel costs^h^, [range]			£7.63 (US $10.39) [£4.57-£13.02 (US $6.22-US $17.73)]		£10.78^g^ (US $14.68) [£4.88-£18.37 (US $6.65-US $25.01)]		<.001^e^
	**Total appointments (N=119), n (%)**				78 (65.5)		41 (34.5)		
		Total distance traveled^f^, miles			2382		2521^g^		N/A^b^
		Total estimated travel time^f^, hours			86.7		69.9^g^		N/A^b^
		Travel costs			£574.04 (US $781.73)		£441.48^g^ (US $601.20)		N/A^b^
	Carbon footprint (CO_2_e)^i^, tonne				0.46		0.49^g^		N/A^b^

^a^Two patients switched from face-to-face to teleconsultation after first visit.

^b^Statistical test not applicable.

^c^Chi-square test.

^d^Independent *t* test (2-tailed).

^e^Mann-Whitney test.

^f^Estimated by Google Maps based on the return journey to the hospital from their home address.

^g^Indicative cost, time, and travel miles avoided by the teleconsultation group.

^h^Based on the average fuel price of £1.28 (US $1.74) per liter during the pilot, 51.7 mpg fuel efficiency, and hospital parking charge of £4 (US $5.45).

^i^Calculated assuming the same fuel efficiency and total mileage of all trips.

### Questionnaire Feedback

#### Teleconsultation Group

**Figure 2:**
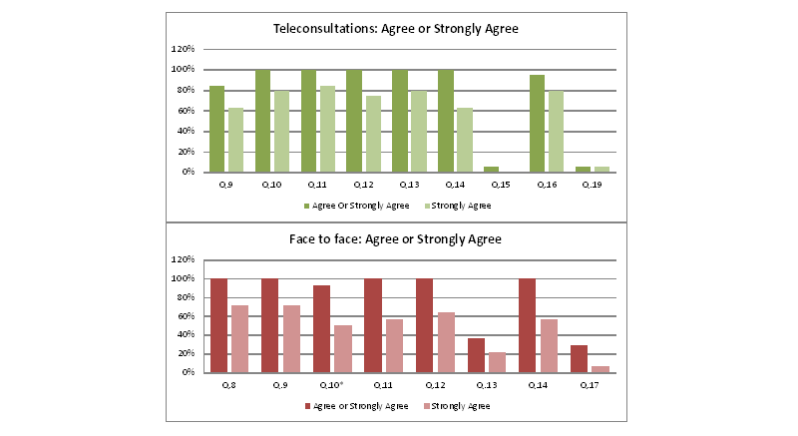
Satisfaction responses in teleconsultation and face-to-face appointment groups.

Of the 24 attendees who participated in teleconsultations, 20 (83%) returned the questionnaire. One respondent gave answers only up to question 8; therefore, there were 19 complete surveys. Laptops or PCs (8/20,40%) and smartphones (8/20, 40%) were the most commonly used devices for participants, with tablets (4/20, 20%) also being used. Home broadband was used in the majority of cases with only 1 participant using mobile broadband. Furthermore, 3 respondents reported difficulty in seeing or hearing during the consultation. One patient reported that they felt physical examination was important (question 15), whereas 74% (14/19) disagreed with the statement and a further 21% (4/19) had no opinion. There was no self-reported cost associated with teleconsultations. Self-reported consultation time, taking the set up into account, was less than 30 minutes in 90% of cases and less than an hour in all cases. When asked if they would rather come in person in the future (question 19), only 1 person stated a preference to come in face-to-face next time. Participant satisfaction responses are shown in Figure 2*.* The travel method and total appointment duration time (including associated travel) of respondents are available in Table 2*.*

**Table 2:** Results of feedback questionnaires.

Question subject		Teleconsultation appointments (n=20)	Face-to-face appointments (n=14)
**Sex, n (%)**			
	Male	10 (50)	7 (50)
	Female	10 (50)	7 (50)
**Travel method (if had to attend), n (%)**			
	Family or friends	15 (75)	12 (86)
	Other (hospital or public transport)	5 (25)	2 (14)
**Total appointment duration (including travel or set up time), n (%)**			
	<30 minutes	18 (90)	0 (0)
	30 minutes to 1 hour	2 (10)	2 (14)
	1-2 hours	0 (0)	4 (29)
	2-3 hours	0 (0)	5 (36)
	3-4 hours	0 (0)	1 (7)
	>4 hours	0 (0)	2 (14)

If participants stated a preference for teleconsultations, they were asked to rank potential reasons (time saving, cost saving, travel saving, minimization of disruption to family life, and prefer teleconsultations over face to face) for this in order of importance (1 being the highest importance and 5 being the least important). Of the 18 who provided answers, the reasons marked *1* or *2* most commonly were time saving (n=12), minimization of disruption to family life (n=11), and travel saving (n=9) as the most important. Cost saving (n=3) and the preference for teleconsultations (n=1) were the least important to patients.

Question 21, concerning issues to improve, was used by participants to share their thoughts regarding teleconsultations. Several respondents shared that there is stress and cost attached to hospital appointments and that the teleconsultations had improved this—a retired couple traveling from 50 miles away said:

Wonderful to be able to speak to specialists in the privacy of our own home. This relieves the stress attached to travelling to and attending clinic. Thank you for offering this service. It would have cost us £50-£60 to attend.

Another commented:

No, the video link has been instrumental in reducing stress levels for face to face clinical appointments. It would have cost £25-30 to attend in person on the stressful nature of face to face clinics and felt teleconsultations had reduced stress, as well as saving money.

One participant, aged 88 years and the main carer for his wife, said, “I felt that this was an excellent way to have an appointment and enabled me to continue caring for my wife as well as receive expert care.” Another added that it was “much more convenient.” Another commented on the suitability of different clinical scenarios that: “It was fine for a routine follow up.”

Any comments that suggested areas for improvement were regarding difficulties receiving the email appointment link or problems with internet speed:

The only problems encountered were due to my signal issues. However I have had difficulties receiving the 'joining' instructions email.

The email with regard to the clinic could only be accessed on my laptop. It never was received on my iPhone or iPad. Apple seemed to have blocked it.

In these cases, the email appointment with the joining link was marked as spam by the recipient’s email carrier. Once the email was retrieved from the spam folder and marked as *safe* by the participant, no further issues were encountered.

#### Face-to-Face Group

Of the 47 participants that attended, 14 (30%) questionnaires were returned. Most (12/14, 86%) patients got to the hospital with the help of family or friends, with 14% (2/14) depending on hospital transport. Self-reported consultation time, taking into account the travel time, was understandably longer with only 14% (2/14) respondents reporting that their appointment from leaving home to returning would take less than 1 hour. In total, 57% (8/14) respondents expected it would take more than 2 hours with two of those expecting to spend more than 4 hours away from home.

As with the teleconsultation survey, user satisfaction was high with all patients reporting satisfaction with their appointment. As with teleconsultations, the proportion of patients who felt physical examination was important was low (5/14, 36%), with most declaring *no opinion* (8/14, 57%). In addition, 4 patients expressed an interest in having a video consultation, with 57% (8/14) preferring to come face to face. All these patients cited a preference for, or a belief that face-to-face appointments were better than teleconsultations.

A total of 10 patients self-reported costs of attendance, which ranged from £5 (US $6.78) to £40 (US $54.27); 80% (8/10) reported costs of at least £10 (US $13.57), whereas 30% (3/10) reported costs of at least £20 (US $27.14). Only 20% (2/10) patients felt that there was no financial cost to attend, one of whom attended hospital transport, whereas the other came with a friend. When asked about improvements that could be made, only 1 person responded stating that “car parking and directions to the department” could be improved.

## Discussion

### Principal Findings

The use of teleconsultations in the United Kingdom has a relatively long history, with studies dating back to the mid-1990s [24]. Technological advances mean that many of the early studies in the United Kingdom relied on expensive audio-visual systems, which made them impractical for outpatient care, especially in a cost-conscious public health service [25]. The Office of National Statistics figures reported that internet access (90% of households) and smartphone usage (78% of adults) is now extremely high throughout the population and rising steadily every year [26]. This means that video calling technology is now accessible to millions of potential patients. Nuffield trust found that public willingness to use video consultations for a variety of medical conditions was as high as 63%, which varied little with age [27]. With their Long Term Plan, the NHS has now put digital interactions to the forefront of its plans. A stated goal is to transform the way outpatients work to avoid up to 30 million face-to-face appointments by using digital interactions [2]. A recent scoping review of the current and historical context of teleconsultations concluded that teleconsultations are safe in a broad range of clinical contexts and are generally well received by patients; however, acceptability cannot be presumed and that local evaluation following implementation should be performed to ensure acceptability and safety [15].

This pilot is the first UK-based evidence in a radiotherapy setting and the first described internationally among patients with brain metastases. The limitations that these patients face with respect to travel and the regional nature of our brain metastases service meant that the natural advantages of teleconsultations could be experienced by those who find it most difficult to attend. At present, in the United Kingdom, a criticism of commercial, General Practice based video services available is that they cater to the healthiest patients. Our service was set up specifically considering the limitations of some of the least able in mind.

Over a third of all patients in our pilot took part in a teleconsultation; this is noteworthy as most teleconsultation services typically report uptake rates of less than 20%. In fact, rates have been as low as 2% among a diabetic cohort, with the highest rate (20%) reported among postoperative patients with hepatobiliary cancer [14]. The relatively high uptake among patients with cancer in our study and among patients with hepatobiliary cancer compared with published rates in other specialties is perhaps unsurprising as oncology patients spend a significant proportion of their time either in hospital or attending hospital appointments, and patients with brain metastases spend more time in the hospital than patients with metastases elsewhere [28]. The conventional doctrine of teleconsultations is that they are best placed to provide routine clinical care and that more complex situations should be dealt with face to face [14]. However, the high use of such services by patients with cancer alludes to a willingness to challenge such preconceptions. Patients with cancer may receive bad news at any consultation; this did not deter patients from using the teleconsultation service. Further qualitative exploration of patients’ experiences of this medium is underway to explore this in greater depth.

How far the patient lived from the treatment center was a significant factor in their decision to participate in teleconsultations. This is supported by the reasons chosen by patients with time saving and less disruption to family life being most important to them. The patient feedback alludes to the stress associated with traveling to the hospital. This is consistent with the findings of a recent report that found that 20% of older patients find simply traveling to hospital stressful [29].

There is a potential for bias in allowing patients to choose their own appointment type (ie, patients will choose it because they think it will be better for them). The rationale behind this is well rooted in the literature with consistent findings from patients that the choice should remain with the patient and that teleconsultations should be offered as an alternative rather than a replacement for face-to-face appointments [14,15,30].

This service evaluation suggests that teleconsultations were acceptable among this group of patients. Satisfaction with the brain metastases service was universally high and this did not differ in the teleconsultation group. Patients felt that nothing was missed (question 12) as their consultant was able to provide satisfactory care (question 13) and that privacy and confidentiality (question 14) were maintained. Interestingly, patients in both groups felt that physical examination was not important for their consultation, which is in contrast to other studies where a lack of physical examination was cited as a concern among patients [17]. Humer et al [31] pointed out that the patient perceived the importance of physical examination may be overstated. An interesting perspective on physical examination is that it has become a ritual done to satisfy the basic needs of patients to feel cared for and for physicians to feel like their work is meaningful [32].

Patients largely self-selected themselves for their preferred service; any patient who took part in a teleconsultation did not switch back to face to face afterward, and among the face-to-face attendees, only 2 of the 47 patients subsequently took part in a teleconsultation. This also demonstrates one of the difficulties in using randomized controlled trial methodology for teleconsultations. The NHS rightly suggests that patients should be able to choose how they see their doctor and that teleconsultations should be an alternative rather than a replacement of traditional appointments [9]. This is supported by a recent report from the Royal College of Physicians, which suggests a mix of appointment types as the ideal model of care [4].

Cost analysis aims to attribute cost to the travel and parking associated with the appointments and is likely to be an underestimate because it does not take into account the cost of the family member’s time, including the potential for loss of earnings associated with hospital appointments. Our assumptions are the *best case* scenario. The real estimated cost of attending an outpatient appointment, reported at an NHS conference, was £17.36 (US $23.64) per hour of travel for a face-to-face appointment, £2 (US $2.72) for a telephone interaction, and £1 for a digital interaction [33]. Therefore, the real cost of the face-to-face appointments was £1505.11 (US $2048.67), and the real cost of the teleconsultations was £41 (US $55.80), with a net saving of £1172.46 (US $1596.62) or £28.60 (US $38.95) per appointment.

The environmental impact of patient travel may seem trivial, but the NHS has a carbon footprint of 22.8 million tons of CO_2_ per year or 6% of the total carbon footprint of the United Kingdom, of which nearly 10% is attributed to travel [34].

### Limitations

A limitation of our work is that we did not include health care provider–associated costs. As this was a pilot study with relatively small numbers of patients, it was not thought to be meaningful data. A larger study would provide better data for this.

We have not collected socioeconomic data on this cohort; therefore, we are unable to draw any conclusions in relation to patient education status, income, and professional status. We have not looked at feedback from health care professionals owing to the small number of clinicians involved. The population involved has disease-specific transport limitations, and although these may apply to other ailments, the results of this pilot may not be generalizable to other services.

### Conclusions

This pilot has demonstrated that teleconsultations in this selected oncology population with travel limitations are popular and acceptable. These benefits may be seen among other patients with cancer, and we plan further pilots in other aspects of cancer care. Further qualitative exploration of participants’ experiences is underway to explore some of the issues raised in this paper in greater depth. The expansion and development of this teleconsultation service is underway, which will allow for further evaluation studies to assess the wider implications of more integrated teleconsultation use in the NHS.

## Appendix

Multimedia Appendix 1 Feedback questionnaires.

## Abbreviations

NHS: National Health Service
SRS: stereotactic radiosurgery

## References

[1] Five year forward view. National Health Service.

[2] NHS Long Term Plan. National Health Service.

[3] (2014). Personalised health and care 2020 using data and technology to transform outcomes for patients and citizens a framework for action 2014. National Information Board and Department of Health and Social Care.

[4] Isherwood M, Hillman T, Goddard A (2018). Outpatients: the future adding value through sustainability. Royal College of Physicians.

[5] Hawkes N (2013). Hospitals without walls. BMJ.

[6] Elrod JK, Fortenberry JL (2017). The hub-and-spoke organization design: an avenue for serving patients well. BMC Health Serv Res.

[7] McLendon SF (2017). Interactive video telehealth models to improve access to diabetes specialty care and education in the rural setting: a systematic review. Diabetes Spectr.

[8] Sood S, Mbarika V, Jugoo S, Dookhy R, Doarn CR, Prakash N, Merrell RC (2007). What is telemedicine? A collection of 104 peer-reviewed perspectives and theoretical underpinnings. Telemed J E Health.

[9] (2015). Technology enabled care services 2015: Resource for Commissioners. NHS Commissioning Assembly.

[10] Lamba N, Muskens IS, DiRisio AC, Meijer L, Briceno V, Edrees H, Aslam B, Minhas S, Verhoeff JJC, Kleynen CE, Smith TR, Mekary RA, Broekman ML (2017). Stereotactic radiosurgery versus whole-brain radiotherapy after intracranial metastasis resection: a systematic review and meta-analysis. Radiat Oncol.

[11] (2016). Patients benefiting from advanced brain tumour treatment set to double. National Health Service.

[12] Bentley R, O'Cathail Micheal, Aznar-Garcia Luis, Crosby V, Wilcock A, Christian J (2019). Defining patterns of care in the management of patients with brain metastases in a large oncology centre: A single-centre retrospective audit of 236 cases. Eur J Cancer Care (Engl).

[13] (2016). Neurological disorders: assessing fitness to drive. Driver and Vehicle Licensing Agency.

[14] Greenhalgh T, Shaw S, Wherton J, Vijayaraghavan S, Morris J, Bhattacharya S, Hanson P, Campbell-Richards D, Ramoutar S, Collard A, Hodkinson I (2018). Real-world implementation of video outpatient consultations at macro, meso, and micro levels: mixed-method study. J Med Internet Res.

[15] O'Cathail Micheal, Sivanandan M, Diver C, Patel Poulam, Christian Judith (2020). The use of patient-facing teleconsultations in the National Health Service: scoping review. JMIR Med Inform.

[16] Taylor M, Khoo K, Saltman D, Bouttell E, Porter M (2007). The use of telemedicine to care for cancer patients at remote sites. JCO.

[17] Sabesan S, Simcox K, Marr I (2012). Medical oncology clinics through videoconferencing: an acceptable telehealth model for rural patients and health workers. Intern Med J.

[18] Thaker DA, Monypenny R, Olver I, Sabesan S (2013). Cost savings from a telemedicine model of care in northern Queensland, Australia. Med J Aust.

[19] Chan BA, Larkins SL, Evans R, Watt K, Sabesan S (2015). Do teleoncology models of care enable safe delivery of chemotherapy in rural towns?. Med J Aust.

[20] (2019). The Price of Fuel. PetrolPrices.

[21] (2017). Energy and environment: data tables. Statistical data set.

[22] Brain J, Schofield J, Gerrish K, Mawson S, Mabbott I, Patel D (2011). A guide for clinical audit, research and service review. Healthcare Quality Improvement Partnership.

[23] Finch T, May C, Mair F, Mort M, Gask L (2003). Integrating service development with evaluation in telehealthcare: an ethnographic study. BMJ.

[24] Darkins A, Fisk N, Garner P, Wootton R (1996). Point-to-point telemedicine using the ISDN. J Telemed Telecare.

[25] Loane M, Bloomer S, Corbett R, Eedy D, Hicks N, Lotery H, Mathews C, Paisley J, Steele K, Wootton R (2000). A comparison of real-time and store-and-forward teledermatology: a cost-benefit study. Br J Dermatol.

[26] (2018). Internet access: households and individuals, Great Britain - Office for National Statistics 2018. Home internet and social media usage.

[27] (2018). The NHS at 70: What will new technology mean for the NHS and its patients?. The King Fund Publications.

[28] Girard N, Cozzone D, de Leotoing L, Tournier C, Vainchtock A, Tehard B, Cortot AB (2018). Extra cost of brain metastases (BM) in patients with non-squamous non-small cell lung cancer (NSCLC): a French national hospital database analysis. ESMO Open.

[29] (2017). Painful Journeys. Age UK: In-Depth Policy Report.

[30] Gilbert AW, Jaggi A, May CR (2018). What is the patient acceptability of real time 1:1 videoconferencing in an orthopaedics setting? A systematic review. Physiotherapy.

[31] Humer MF, Campling BG (2017). The role of telemedicine in providing Thoracic Oncology Care to remote areas of British Columbia. Curr Oncol Rep.

[32] Costanzo C, Verghese A (2018). The physical examination as ritual: social sciences and embodiment in the context of the physical examination. Med Clin North Am.

[33] Bernard Quinn (2018). Digital technology transformation of outpatient services?. Health & Medicine.

[34] (2018). Natural Resource Footprint. National Health Service - Sustainable Development Unit.

