# Supplementary material for: Use of Teleconsultations in a Regional Stereotactic Radiosurgery Service: Pilot Study
Source: JMIR Form Res. 2021 Feb 5;5(2):e15598. doi: 10.2196/15598 (PMC7895639; doi:10.2196/15598)
Supplement: Multimedia Appendix 1 [file formative_v5i2e15598_app1.docx]

**Teleconsultation feedback quetsionnaire**

**1. What is your Gender?**

**2. What is your age? (years)**

**3. What type of clinic visit was this?**

**4. How far from Nottingham City Hospital do you live?**

**5. If you had to travel to the hospital for your appointment, how would you have gotten there?**

**6. How did your long video appointment take?**

**7. What type of device did you use for your video consultation?**

**8. What type of internet connection did you use?**

**9. I had no difficulty seeing and hearing the doctor through the video link.**

**10. I could talk to the specialist easily and openly.**

**11. I felt I could ask my specialist questions.**

**12. I did not feel anything important was missed during my visit with my doctor.**

**13. I felt my consultant was able to understand my situation and provide satisfactory care**

**14. I felt my privacy and confidentiality were preserved during my visit with the doctor.**

**15. I felt it was important for the specialist to examine me.**

**16. Overall I was satisfied with the appointment today.**

**17. Can you estimate how much attending this appointment cost you? (Consider how much you have spent on travel/fuel, parking and any other expenses you had)**

**18. Have you ever had to cancel appointments because you were unable attend due to the following?**

**19. I would rather travel to Nottingham to see my specialist than participate in a video consultation again.**

**20a. If you indicated your preference for face to face appointments, could you please tell us why?**

**20b. If you indicated your preference for video clinics, could you please tell us why?**

**21. Is there anything you feel important that we need to address to improve?**

**Face-to-face feedback quetionaire**

**1. What is your Gender?**

**2. What is your age?**

**3. What type of clinic visit was this?**

**4. How far from Nottingham City Hospital do you live?**

**5. How have you travelled to hospital for your appointment?**

**6. From when you left home to when you return how much time do you estimate your appointment will take you? (Please include any WAITING and TRAVEL to and from your usual residence)**

**7. If you have come with family/friends, did they have to take time off work to come with you?**

**8. I could talk to the specialist easily and openly.**

**9. I felt I could ask my specialist questions.**

**10. I did not feel anything important was missed during my visit with my doctor.**

**11. I felt my specialist was able to understand my situation and provide satisfactory care**

**12. I felt my privacy and confidentiality were preserved during my visit with the doctor.**

**13. I felt it was important for the specialist to examine me.**

**14. Overall I was satisfied with the appointment today.**

**15. Can you estimate how much attending this appointment cost you? (Consider how much you have spent on travel/fuel, parking and any other expenses you had)**

**16. Have you ever had to cancel appointments because you were unable attend due to the following? (Tick all that apply)**

**17. The department is offering the opportunity for patients to see your specialist by a video consultation (like Skype or Face Time) instead of attending face to face. Would you be interested in taking up this offer for future appointments?**

**18. If you indicated your preference for face to face appointments, could you please tell us why?**

**19. Is there anything you feel important that we need to address to improve?**
